# Molecular Detection of *Cryptosporidium cuniculus* in Rabbits (*Oryctolagus cuniculus*) from Tenerife, Canary Islands, Spain

**DOI:** 10.3390/vetsci9020091

**Published:** 2022-02-18

**Authors:** Edgar Baz-González, Natalia Martín-Carrillo, Katherine García-Livia, Pilar Foronda

**Affiliations:** 1 Department Obstetricia y Ginecología, Pediatría, Medicina Preventiva y Salud Pública, Toxicología, Medicina Legal y Forense y Parasitología, Universidad de La Laguna, 38200 San Cristóbal de La Laguna, Tenerife, Canary Islands, Spain; alu0100814583@ull.edu.es (E.B.-G.); nataliamartincarrillo@gmail.com (N.M.-C.); kathegl16@gmail.com (K.G.-L.); 2 Instituto Universitario de Enfermedades Tropicales y Salud Pública de Canarias, Universidad de La Laguna, 38200 San Cristóbal de La Laguna, Tenerife, Canary Islands, Spain

**Keywords:** *Cryptosporidium cuniculus*, zoonoses, *Oryctolagus cuniculus*, rabbits, Canary Islands, Spain

## Abstract

*Cryptosporidium cuniculus* is a zoonotic parasite responsible for cryptosporidiosis cases and outbreaks in both humans and rabbits. Since there are no molecular *Cryptosporidium* spp. infection data in rabbits (*Oryctolagus cuniculus*) from Spain, our aim was to gather information about this parasite in wild European rabbits from Tenerife, Canary Islands (Spain). A total of 100 faecal samples were collected from rabbits from eight municipalities of Tenerife. Microscopic analysis showed that 4.0% of the samples presented structures compatible with *Cryptosporidium* oocyst. A nested polymerase chain reaction (PCR) targeting *18S* ribosomal RNA (rRNA) gene fragments was carried out, and sequencing confirmed the identity of *C. cuniculus* in one sample (1.0%). The sample was successfully subtyped using nested PCR analysis of the 60-kDa glycoprotein (*gp60*) gene as the subtype VbA26R3. This study confirms the presence of *C. cuniculus* in wild rabbits from Tenerife, providing new information on the occurrence of this zoonotic parasite. Further studies are required to better understand the epidemiology of *Cryptosporidium* spp. in wild rabbits in Spain and their possible public health repercussions.

## 1. Introduction

The Canary Islands are a Spanish archipelago composed of eight islands and five islets located in the Atlantic Ocean, near the coast of northwest Africa (13°23′–18°8′ W and 27°37′–29°24′ N). The European rabbit (*Oryctolagus cuniculus*), an “invasive introduced” species [1] native to the Iberian Peninsula, was introduced to these islands in the 15th century [1]. Nowadays, it has been established as a species of economic and cultural interest in the islands, due to hunting activity and farming. The Canary Islands have 3.2% of the rabbit farms of Spain [2] and the wild rabbit is one of the small game hunting species in Tenerife [1]. In 2017, rabbit abundance was estimated in a mean value of 2.22 individuals/ha in Tenerife, with a standard derivation of 2.25 individuals/ha, which suggests that there is high spatial variability in the abundance of the species. In general, abundance was higher in areas of low elevation and slope [3].

The European rabbit is a well-known host of several pathogens such as helminths, viruses, and protozoa [4], including *Cryptosporidium* spp.

*Cryptosporidium* is a genus of parasitic protozoa comprising of 48 valid species and more than 100 genotypes [5] that infect a wide range of hosts, including humans, mammals, birds, reptiles, and fish [6]. The transmission of the infective stage occurs through the ingestion of sporulated oocysts via contact with an infected person/animal, ingestion of contaminated water or food, or possibly through the air [7]. Infection ranges from being asymptomatic to mild–severe diarrhea. The parasite has a cosmopolitan distribution, particularly in developing countries [8].

Since *Cryptosporidium* spp. infection was first detected in a rabbit in 1912 by Tyzzer [9], other cases in this mammal have been reported, most of them caused by *Cryptosporidium cuniculus* (previously known as *Cryptosporidium* rabbit genotype), which first identified using transmission electron microscopy in 1979 [10], and was morphologically and molecularly re-described and characterized in 2010 [11]. The notable countries of *C. cuniculus* infection in rabbits are the USA [10], China [12,13,14,15,16,17,18], the Czech Republic [19], New Zealand [20], the United Kingdom (UK) [21], Australia [22,23,24,25,26], Poland [27], Brazil [28], and Egypt [29]. Although *C. cuniculus* is the most frequent species detected in rabbits, *Cryptosporidium fayeri* and *Cryptosporidium parvum* have also been reported in this lagomorph in Australia and Nigeria, respectively [26,30].

To date, the known hosts for *C. cuniculus* are humans, rabbits (*O. cuniculus*), Eastern grey kangaroos (*Macropus giganteus*) [31], and more recently, alpacas (*Vicugna pacos*) have been studied as possible host [32]. It has also been found in wastewater treatment plants in the UK [21,33], China [34,35], Brazil, Peru [36], Spain [37], and Australia [38,39]; in rivers in China [40], Australia [41], and South Africa [42]; and sources of water in Australia [43,44].

In humans, the most common etiologic agents of cryptosporidiosis are Cryptosporidium hominis and Cryptosporidium parvum [45], while Cryptosporidium canis, Cryptosporidium cuniculus, Cryptosporidium erinacei, Cryptosporidium felis, Cryptosporidium meleagridis, Cryptosporidium tyzzeri, and Cryptosporidium ubiquitum are associated with a lower number of cases [46]. C. cuniculus has been reported as a confirmed agent of human cryptosporidiosis in (see Table 1) the UK [21,47,48,49], France [50,51,52], Nigeria [53], Australia [31], New Zealand [54,55,56], Canada [57], Sweden, and Greece [58]. To date, the only two cases linked to Spain are the case of a symptomatic pediatric patient from Madrid [59] and a Spanish travel-related case [58].

In the Canary Islands, several *Cryptosporidium* species/genotypes have been reported: in patients from Tenerife [60], Gran Canaria, Lanzarote, and La Palma [61,62]; in pigeons (*Columba livia*) from Gran Canaria and Tenerife [63,64]; in rodents from Tenerife, La Palma, and El Hierro [65,66]; and in hedgehogs (*Atelerix algirus*) [67] and wastewater from Tenerife [68,69]. Since there is no infection data in rabbits in the islands, the aim of the present study was to screen a wildlife population of rabbits from Tenerife for the presence of *Cryptosporidium* spp. with staining and molecular methods.

## 2. Materials and Methods

### 2.1. Sample Collection

**Figure 1:**
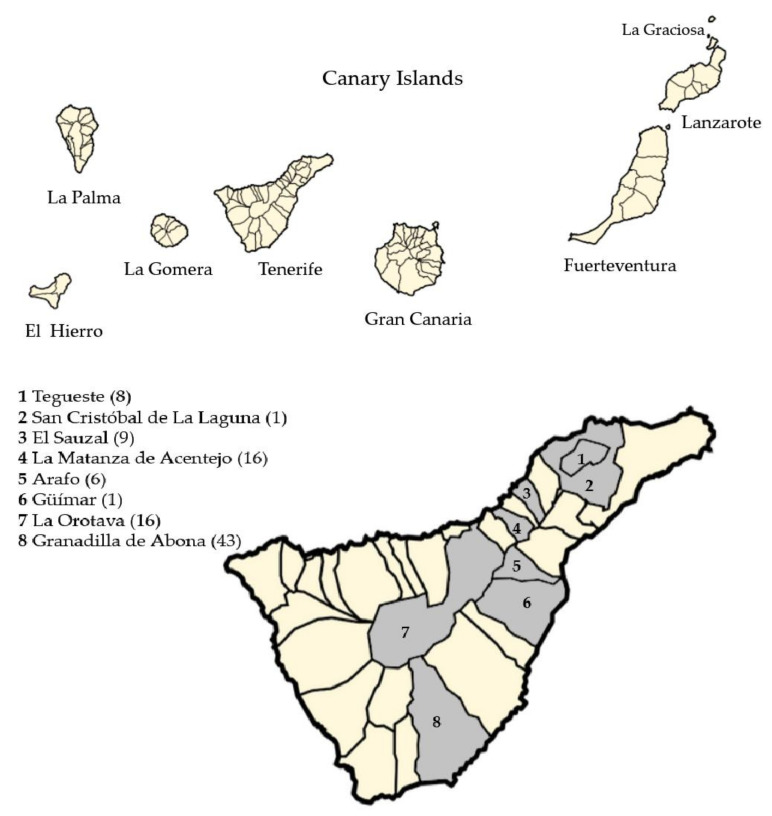
The sampling area. Map of the Canary archipelago and the island of Tenerife showing in grey zones the sampled municipalities. Inserted in brackets are the number of samples/municipality (The original images were taken from Wikimedia Commons (https://commons.wikimedia.org/w/index.php?title=File:Mapa_Canarias_municipios.svg&oldid=478721455, accessed on 11 February 2022; https://upload.wikimedia.org/wikipedia/commons/d/d1/Mapa_Canarias_municipios.svg, accessed on 11 February 2022; https://commons.wikimedia.org/wiki/File:Mapa_Canarias_municipios.svg, accessed on 11 February 2022; https://upload.wikimedia.org/wikipedia/commons/d/d1/Mapa_Canarias_municipios.svg, accessed on 11 February 2022) in which the permission to copy, distribute, or adapt it is established. User: Júlio Reis (https://commons.wikimedia.org/wiki/User:Tintazul accessed on 11 February 2022). The images were edited by the Paint 3D program).

A total of 100 faecal samples from rabbits donated by hunters (*n* = 82) and found dead (*n* = 18), from eight municipalities of Tenerife: Tegueste, San Cristóbal de La Laguna, El Sauzal, La Matanza de Acentejo, Arafo, La Orotava, Güímar, and Granadilla de Abona (Figure 1) were collected between 2015–2017 and placed into sterile plastic containers until reception in the laboratory, then were subsequently deposited in vials containing 2.5% aqueous (*w*/*v*) potassium dichromate (K_2_Cr_2_O_7_) solution. The samples were stored at 4 °C until the posterior analysis.

### 2.2. Ethical Statement

The samples used in this study were donated by hunters that hunted wild rabbits during the legal hunting season period, detailed in the numbers of the Official Bulletin of Canaries (BOC) (http://www.gobiernodecanarias.org/boc/ accessed on 11 February 2022); 128 from 2015, and 125 from 2016 and 2017, respectively. Therefore, no rabbits were sacrificed for this study. No ethical approval was required.

### 2.3. Staining Method

Samples were stained using the Kinyoun TB Stain Kit K (Becton, Dickinson and Company, USA) following the manufacturer’s instructions and microscopically screened for *Cryptosporidium* spp. oocysts. The samples with oocyst-compatible structures were identified as positive and preserved until DNA extraction and PCR analysis.

### 2.4. DNA Extraction

An aliquot of ∼200 µL of each sample identified as positive diluted in potassium dichromate was washed with PBS-EDTA at room temperature to remove the potassium dichromate. Then, they were transferred to centrifuge tubes containing 500 µL of lysis buffer, and one freeze-thaw cycle (−80 °C to +100 °C) in boiling water was made prior the extraction procedure [70]. Total DNA was isolated with the commercial FastDNA^®^ Spin Kit for Soil (MP Biomedicals, Solon, OH, USA) following the manufacturer’s instructions, with the homogenizer FastPrep-24™ 5G (MP Biomedicals, Solon, OH, USA) as oocyst disruptors.

### 2.5. PCR Amplification

Two *Cryptosporidium* spp. genes fragments were amplified by nested PCR (Table 2). The first one was performed targeting an 830 bp nucleotide fragment of the *18S* ribosomal RNA (rRNA) gene, using the primers SSU-F1/SSU-R1 for the primary and SSU-F2/SSU-R2 for the secondary reactions [71]. The reaction mixture in both steps of the nested PCR contained 0.125 µL of Taq DNA polymerase (5 U/µL) (VWR), 1 µL of each primer (10 µM), 2.5 µL of dNTPs mix (200 µM) (Bioline, London, UK), 1 µL MgCl_2_ (25 mM) (VWR), 2.5 µL 10× key buffer (15 mM Mg^2+^) (VWR), 2 µL of DNA template (or 2 µL of primary PCR product for the secondary PCR), and water, to a total volume of 25 µL.

The subtype of the positive samples was detected targeting an 800–850 bp nucleotide fragment of the 60-kDa glycoprotein (*gp60*) gene, using the primers AL3531/AL3535 for the primary, and AL3532/AL3534 for the secondary PCRs [72]. The reaction mixture contained 0.125 µL of Taq DNA polymerase (5 U/µL) (VWR), 0.5 µL of each primer (10 µM), 2.5 µL of dNTPs mix (200 µM) (Bioline, London, UK), 1.5 µL MgCl_2_ (25 mM) (VWR), 2.5 µL 10× key buffer (15 mM Mg^2+^) (VWR), 1 µL of DNA template (or 1 µL of primary PCR product in the secondary PCR), and water, to a total volume of 25 µL.

PCR reactions were performed in a XP Cycler (Bioer Technology, Hangzhou, China) thermocycler and were visualized on 1.5% (*w*/*v*) agarose gels (Fisher Bioreagents, Madrid, Spain) stained with REALSAFE Nucleic Acid Staining Solution (20,000×, REAL, Durviz S.L., Valencia, Spain).

The nested PCR products were sequenced at Macrogen Spain, with the secondary pair of primers in both senses.

### 2.6. Sequencing and Phylogenetic Analysis

The fragments of the nucleotide sequences obtained were edited with the MEGA X program [73], and subsequently aligned with other *Cryptosporidium* species/subtypes sequences using the ClustalW program included in MEGA X. Minor corrections, to increase the aligned sequence similarity and improve the inferences on any positional homology, were then made by hand.

A Basic Local Alignment Search Tool (BLAST) search was carried out in order to elucidate any homologies or similarities with the sequences previously published in the GenBank database.

The molecular identification of the *18S* rRNA and *gp60* genes was achieved by phylogenetic analysis through the Neighbor-Joining distance method with the p-distance model [74] and maximum-likelihood method with a Tamura–Nei model [75], both with at least 1000 bootstrap replications in MEGA X using the sequence of *Eimeria magna* (HQ173833.1) as the outgroup. Nucleotide sequences obtained in this work were submitted to the GenBank database under the accession numbers OM170342 and OM249938 for *18S* rRNA and *gp60* genes, respectively.

## 3. Results

### 3.1. Staining and Molecular Results

In four (4.0%) of the faecal samples screened by the Kinyoun method and light microscopy, *Cryptosporidium* oocyst-compatible structures were found. One sample was amplified by nested PCR with the expected size, and it was identified as *C. cuniculus* by sequencing.

Therefore, the occurrence of *C. cuniculus* in wild rabbits from Tenerife was 1.0% (1/100). The positive sample was located in La Orotava from a rabbit donated by hunters, with an overall occurrence of 6.25% (1/16) in this municipality.

### 3.2. Phylogenetic Analyses

#### 3.2.1. *18S* rRNA Gene Analysis

A fragment of 762 bp was obtained for the *18S* rRNA gene of *C. cuniculus*. The BLAST showed highest homology with various *C. cuniculus* isolated (Acc. Number: MH341587.1; MG516742.1; KT336619.1; KY483978.1, Query Cover: 100%, Identity: 99.87%).

**Figure 2:**
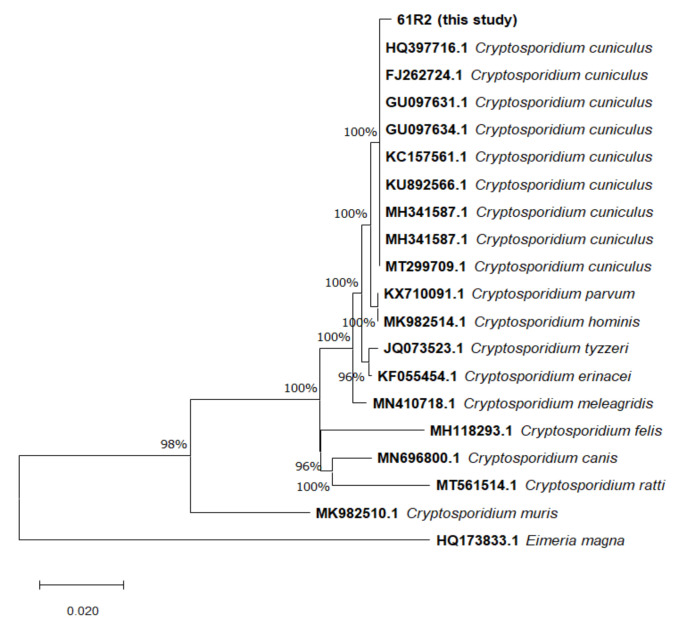
Neighbor-Joining tree of *Cryptosporidium* spp. based on the *18S* rRNA gene sequence.

An alignment of 798 bp was used for the phylogeny. The result of the Neighbor-Joining analysis based on the *18S* rRNA gene is shown in Figure 2.

#### 3.2.2. *gp60* Analysis

A fragment of 798 bp was obtained for the *gp60* gene of *C. cuniculus*.

The BLAST analysis showed highest homology with *C. cuniculus* VbA26subtype (Acc. Number: MT265707.1, Query Cover: 100%, Identity: 99.87%), and VbA27 (Acc. Number: KY123920.1, Query Cover: 100%, Identity: 99.63%), both isolated from humans in New Zealand.

**Figure 3:**
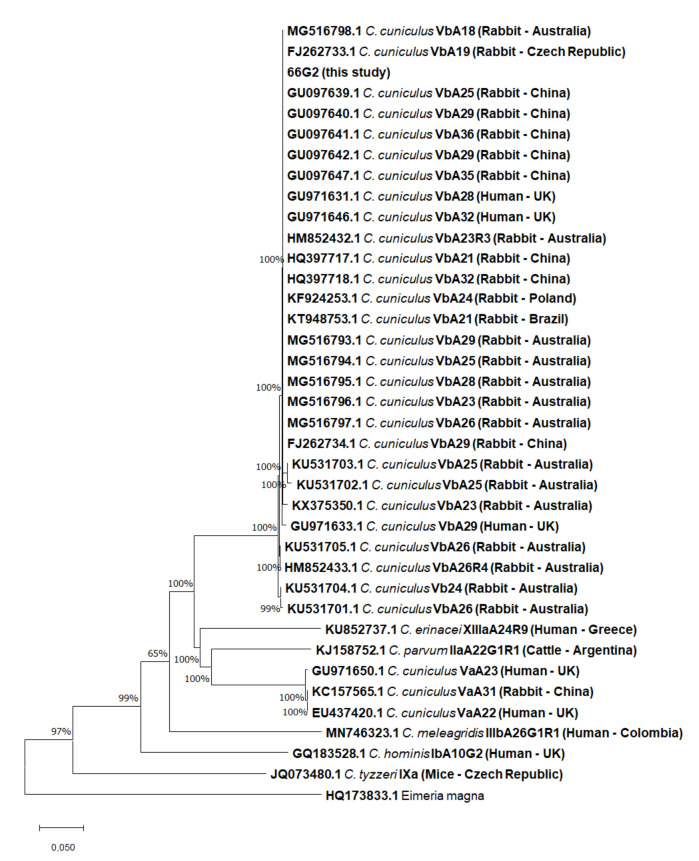
Neighbor-Joining tree of *Cryptosporidium* spp. genotypes based on *gp60*. Each sequence is identified by its accession number, species, and subtype, and inserted in brackets are the host and country of origin.

An alignment of 921 bp was used for the phylogeny. The result of the Neighbor-Joining analysis based on *gp60* is shown in Figure 3.

The results of the Neighbor-Joining (Figure 2 and Figure 3) and the maximum-likelihood (Supplementary Material, Figures S1 and S2) analyses based on the *18S* rRNA and *gp60* genes identified and isolated as *C. cuniculus* (100% bootstrap) clearly separated to other *Cryptosporidium* species (Figure 2), belonging to the Vb family (100% bootstrap) and differing from the Va family (Figure 3), respectively.

## 4. Discussion

The present study provides data about the occurrence of *Cryptosporidium cuniculus* in wild rabbits in the Canary Islands. To date, some previous studies carried out in rabbits were based on the detection of *Cryptosporidium* spp. without reaching the species level due to the techniques employed: faecal examination using light microscopy in pet rabbits in Japan [76], Brazil [77] and Egypt [78]; immunofluorescent microscopy in faecal samples from pet rabbits in the UK [79]; antigen detection by enzyme-linked immunosorbent assay (ELISA) in farmed rabbits in Nigeria [80]; and staining methods in rabbits from Spain [81,82], Australia [83], the UK (see [84]), China (see [14]), Iraq [85], and Ecuador [86].

Before the re-description of the *C. cuniculus* species, other authors cited *C. parvum* in rabbits, and using light microscopy and indirect immunofluorescence in the UK [87], staining and/or histological techniques in the Czech Republic, Belgium, Hungary, and the USA (see [84]), and staining and molecular methods in China (see [14]) may have led to misdiagnoses.

In the Iberian Peninsula (Spain) from where the Canary rabbit population was introduced, previous studies have already evidenced the presence of *Cryptosporidium* spp. in faecal samples from rabbits: in farmed rabbits from Toledo [81], farmed rabbits from León, and wild rabbits from Madrid [82], but in these cases the specific diagnosis was not possible. To our knowledge, three publications describing the detection of *C. cuniculus* in Spain are available, of which two cases were in humans, one autochthonous and a Spanish travel-related case, in addition to a wastewater detection [37,58,59]. However, the molecular detection of *C. cuniculus* in wild rabbits from Spain had not been reported.

The occurrence of *C. cuniculus* in wild rabbits from Tenerife of 1.0% (1/100) is similar to other studies carried out on wild rabbits from Europe, ranging from 0–0.9% in Germany (0/232) and the UK (1/109). These data differ from those reported in wild rabbits from the UK, 7% (2/28); Spain, 100% (7/7) [82], and The Netherlands, 6.5% (2/31) (see [84]); possibly due to the small size of these studies.

Chalmers et al. 2009 [21] propose the classification of the *C. cuniculus* subtypes into two families, Va and Vb, depending on the TCA repetition number in tandem with the microsatellite region of the *gp60* gene, and Nolan et al. 2010 [22] recommend the subtype division depends on ACA triplet repetitions in the region immediately following the microsatellite region, specifying it with an R. Following the proposed nomenclature, the novel genotype sequence obtained in this study (Acc. Number: OM249938) should be named VbA26R3.

Other authors cited VbA26subtype detection in wild rabbits from Australia [23,24,26], in Eastern grey kangaroos [24,31], in South Africa river water [42], and humans from the UK [48] and New Zealand [55], thus confirming the zoonotic potential of this subtype.

There are previous studies on molecular epidemiology in wild rabbits, but this was only located in Australia, with prevalence data ranges from 2.18–14.3%, and with the Vb family being the only family detected and VbA23subtype being the most prevalent (Table 3), highlighting *C. cuniculus* as the most prevalent *Cryptosporidium* species reported in more than 11 host species in wildlife in Australia [23,24]. Outside Australia, there is only molecular epidemiology data of farmed rabbits, with prevalence ranging from 2.38–12.73% (Table 3), and the Vb family also being the most common (see Supplementary Material, Table S1: *Cryptosporidium cuniculus* subtypes reported worldwide).

In 2018, Spain was the fourth highest country in the European Union/European Economic Area (EU/EEA) with confirmed cases of cryptosporidiosis [88]. The national cases in 2018 were 1526 (5.22 per 100,000 population) in 11 autonomous communities and the autonomous city of Ceuta (not all of the country is notifying cases). In that year, the Canary Islands had the lowest notification rate of cryptosporidiosis in Spain (0.46 per 100,000 population) [89].

Since 2015, cryptosporidiosis has been declared as a notable disease in Spain. From that year, 54 cases have been confirmed in the Canary Islands, and in that period none of them were in Tenerife (Table 4). Considering the previous detection of *Cryptosporidium* species in Tenerife in humans from 2002–2004 [60], and in wildlife [64,66,67] and wastewater [68,69], possible underdiagnosis may have occurred.

The detection of the zoonotic species *C. cuniculus* in this study highlights the potential role that wild rabbits can play in the maintenance and transmission of this species. Although the majority of rabbit cases develop asymptomatically, the infection can be associated with different pathologies, which can affect farm productivity and even animal survival, leading to economic losses [27,81,90]. On the other hand, while the risk of zoonotic transmission is low, it should not be dismissed since *C. cuniculus* has been involved in human cases (see Table 1) and outbreaks [21], even becoming the third cause of cryptosporidiosis in the UK [48] and New Zealand [56].

For these reasons, further studies are required to better understand the epidemiology of *Cryptosporidium* spp. in wild rabbits in the Canary Islands and their possible public health repercussions.

## 5. Conclusions

The present study constitutes the first molecular detection of *C. cuniculus* in wild rabbits from Tenerife (Canary Islands, Spain), leading to the identification of the VbA26R3 subtype, with potential zoonotic risk. Considering the possible implications to public health of these results, more studies are required in order to evaluate the public health and veterinary risk.

## Figures and Tables

**Table 1:** Human confirmed cryptosporidiosis cases by *Cryptosporidium cuniculus* reported.

Country	Subtype (*n*)	Period	Case/Prevalence (%) (*n*º Positive Cases/Total)	Reference
UK	VaA22 (1)	2007	1 case *	[21,47]
UK	VbA11 (1), VbA20 (1), VbA22 (1), VbA23 (1), VbA25 (1), VbA26 (1), VbA28 (1), VbA29(2), VbA30 (1), VbA32 (1), VbA33 (1), VbA34 (1), VbA36 (1), VbA37 (1), VaA9 (1), VaA18 (1) VaA19 (1), VaA21 (1) VaA22 (1)	2007–2008	1.2% (37/3,030)	[48]
UK	VaA18 (23)	2008	23 cases (422 estimated)	[21,33]
Nigeria	-	2006–2007	6.5% (5/77)	[53]
France	-	2006–2009	0.3% (1/310)	[50]
Australia	VbA25 (1)	2009	1 case *	[31]
Spain	VbA34 (1)	2015	1 case *	[59]
New Zealand	VbA22 (2) VbA25 (1) VbA27 (1)	2009–2015	0.7% (4/579)	[54]
France	-	2015–2017	1% (1/87)	[51]
France	-	2017–2019	>1% (no data)	[52]
New Zealand	VbA13 (2), VbA15 (1) VbA17 (4), VbA22 (3) VbA23 (2), VbA24 (5) VbA25 (8), VbA26 (3) VbA27 (2), VbA28 (3)	2009–2019	1.3% (33/2,598)	[55]
New Zealand	Vb	2015–2021	1.9% (28/1,502)	[56]
Canada	VbA38 (1)	2008–2017	0.8% (1/129)	[57]
Sweden	VaA19 (1) VbA20R2 (1) VbA29R4 (1)	2013–2014	1.3% (5/379)	[58]
Spain	VbA25R3 (1)			
Greece	VbA31R4 (1)			
UK	-	2018–2020	2.8% (3/109)	[49]

* No prevalence data.

**Table 2:** PCR primers and conditions used in this study for *Cryptosporidium* gene amplification.

Target Gene	Primer	Primer Sequences (5′-3′)	Expected Size (bp)	PCR Conditions ^1^
*18S* rRNA	18SF1 18SR1	CCCATTTCCTTCGAAACAGGA TTCTAGAGCTAATACATGCG	830	94 °C—45 s 55 °C—45 s 72 °C—1 min For 35 cycles
	18SF2 18SR2	AAGGAGTAAGGAACAACCTCCA GGAAGGGTTGTATTATTAGATAAAG		94 °C—45 s 58 °C—45 s 72 °C—1 min For 35 cycles
*gp60*	AL3531 AL3535	ATAGTCTCCGCTGTATTC GGAAGGAACGATGTATCT	800–850	94 °C—45 s 50 °C—45 s 72 °C—1 min For 35 cycles (both steps)
	AL3532 AL3534	TCCGCTGTATTCTCAGCC GCAGAGGAACCAGCATC		

^1^ All nested PCR conditions were preceded by 5 min at 95 °C and a final step of 10 min at 72 °C.

**Table 3:** Detection of *Cryptosporidium cuniculus* in rabbits.

**Farmed Rabbits**				
**Country**	**Subtype (*n*)**	**Period**	**Case/Prevalence (%)** **(*n*** **º Positive Cases/Total)**	**Reference**
Czech Republic	VbA19 (1)	-	2 cases *	[19,21]
China	VbA29 (18), VbA35 (4), VbA36 (8)	2007–2008	3.4% (37/1,081)	[13]
China	VbA32 (3), VbA21 (6)	2008–2010	2.38% (9/378)	[14]
Poland	VbA24 (-)	2012	300 cases *	[27]
Brazil	VbA21 (7)	2012	12.73% (7/55)	[28]
China	VbA28 (2) VbA29 (16) VbA32 (3)	2015–2016	11.2% (24/215)	[16]
China	VbA24 (5)	2015–2017	3.4% (11/321)	[17]
Egypt	VbA19 (1) VbA33 (15)	2015–2016	11.9% (28/235)	[29]
China	VbA24 (1) VbA29 (2) VbA31 (2) VbA33 (1)	-	6 isolates*	[18]
**Wild Rabbits**				
**Country**	**Subtype (*n*)**	**Period**	**Case/Prevalence (%)** **(*n*** **º Positive Cases/Total)**	**Reference**
New Zealand	-	2000–2003	1 case *	[20]
UK	VaA18 (1)	2008	1 case *	[21]
Australia	VbA23R3 (11), VbA26R4 (1)	-	6.8% (12/176)	[22]
Australia	VbA22R4 (-) VbA23R3 (-) VbA24R3 (-) VbA25R4 (-) VbA26R4 (-)	2009–2011	8.4% (22/263)	[23]
Australia	VbA25 (2) VbA26 (1) VbA26 novel (3) VbA24 novel (1)	2011–2015	2.18% (7/321)	[24]
Australia	VbA23 (9)	2013–2015	13.2% (14/106)	[25]
Australia	VbA18 (12) VbA23 (46) VbA25 (16) VbA26 (8) VbA28 (2) VbA29 (5)	2013–2015	14.3% (96/672)	[26]
**Others**				
**Country**	**Subtype (*n*)**	**Period**	**Case/Prevalence (%)** **(*n*** **º Positive Cases/Total)**	**Reference**
China (unknown origin)	VbA29 (1)	-	2 cases *	[12,21]
China (animal house)	VaA31 (3)	-	1.03% (3/290)	[15]

* No prevalence data.

**Table 4:** Human-confirmed cryptosporidiosis cases in the Canary Islands (2015–2020) [62].

Year	Cases	Island (*n*)	Notification Rate (per 100,000 Population)	Reference
2015	4	Gran Canaria (4)	0.2	[62]
2016	4	Gran Canaria (2) Lanzarote (2)	0.2	
2017	26	Gran Canaria (25) Lanzarote (1)	1.2	
2018	10	Gran Canaria (9) La Palma (1)	0.5	
2019	7	Gran Canaria (7)	0.3	
2020	3	Gran Canaria (3)	0.1	

## Supplementary Materials

The following supporting information can be downloaded at: https://www.mdpi.com/article/10.3390/vetsci9020091/s1: Figure S1: Maximum-Likelihood tree of *Cryptosporidium* spp. based on the *18S* rRNA gene sequence; Figure S2: Maximum-Likelihood tree of *Cryptosporidium* spp. genotypes based on *gp60* sequences; Table S1: *Cryptosporidium cuniculus* subtypes reported worldwide.
