# Supplementary material for: Molecular Detection of Cryptosporidium cuniculus in Rabbits (Oryctolagus cuniculus) from Tenerife, Canary Islands, Spain
Source: Vet Sci. 2022 Feb 18;9(2):91. doi: 10.3390/vetsci9020091 (PMC8877424; doi:10.3390/vetsci9020091)
Supplement: Supplementary file 1 [file vetsci-09-00091-s001.zip › vetsci-1575771-supplementary(1).pdf]

Table S1.

Table S1. *Cryptosporidium cuniculus* subtypes reported worldwide.

Subtypes	Host	Country	GenBank Accesion Number	Reference
VaA9	Human	UK	GU971640.1	[48]
	River water and Riverbed Sediment	South Africa	-	[42]
VaA11	Riverbed Sediment	South Africa	-	[42]
VaA18	Treated drinking water	UK	FJ262730.1	[21]
	Rabbit	UK	FJ262731.1	[21]
	Human	UK	FJ262732.1	[21]
	Human	UK	GU971636.1	[48]
	River water and Riverbed Sediment	South Africa	-	[42]
VaA19	Human	UK	GU971642.1	[48]
	Human	Sweden	KU852733	[58]
	River water and Riverbed Sediment	South Africa	-	[42]
VaA21	Human	UK	GU971634.1	[48]
	River water and Riverbed Sediment	South Africa	-	[42]
VaA22	Human	UK	EU437420.1	[21,47]
	Human	UK	GU971641.1	[48]
VaA23	Human	UK	GU971650.1	[48]
	Riverbed Sediment	South Africa	-	[42]
VaA31	Wastewater	China	-	[34]
	Rabbit	China	KC157565.1	[15]
	River water and Riverbed Sediment	South Africa	-	[42]
Va	Catchment water	Australia	-	[41]
Vb	Catchment water	Australia	-	[41]
VbA11	Human	UK	GU971637.1	[48]

VbA13	Human	New Zealand	MT265698.1	[55]
VbA15	Human	New Zealand	MT265699.1	[55]
VbA17	Human	New Zealand	MT265700.1	[55]
	Human	New Zealand	MT265701.1	[55]
VbA18	Rabbit	Australia	MG516798.1	[26]
VbA19	Rabbit	Czech Republic	FJ262733.1	[21,19]
	Rabbit	Egypt	MT742540.1	[29]
VbA20	River	China	-	[40]
VbA20R2	Human	Sweden	KU852735	[58]
VbA21	Rabbit	China	HQ397717.1	[14]
	Rabbit	Brazil	KT948753.1	[28]
	Riverbed Sediment	South Africa	-	[42]
VbA22	Human	UK	GU971644.1	[48]
	Human	New Zealand	KY123918.1	[54]
VbA22R4	Rabbit	Australia	KC283002.1	[23]
VbA23	Rabbit	Australia	KX375350.1	[25]
	Rabbit	Australia	MG516796.1	[26]
	Human	New Zealand	MT265702.1	[55]
	Human	New Zealand	MT265703.1	[55]
VbA23R3	Rabbit	Australia	HM852432.1	[22,23]
VbA24	Rabbit	Poland	KF924253.1	[27]
	Rabbit	Australia	KU531704.1	[24]
	Rabbit	China	100% homology with KF924253.1	[17]
	Human	New Zealand	MT265704.1	[55]
	Human	New Zealand	MT265705.1	[55]

	Human	New Zealand	MT265706.1	[55]
	Riverbed Sediment	South Africa	-	[42]
	Rabbit	China	MZ460994.1	[18]
VbA24R3	Rabbit	Australia	KC283005.1	[23]
VbA25	Human	UK	GU971647.1	[48]
	Human	Australia	KM366139.1	[31]
	Rabbit	Australia	KU531702.1	[24]
	Rabbit	Australia	KU531703.1	[24]
	Human	New Zealand	KY123919.1	[54]
	Alpaca	Australia	MH346122.1	[32]
	Rabbit	Australia	MG516794.1	[26]
	Human	New Zealand	MT265694.1	[55]
	Human	New Zealand	MT265695.1	[55]
	River water and Riverbed Sediment	South Africa	-	[42]
VbA25R3	Human	Spain	KU852731	[58]
VbA25R4	Rabbit	Australia	KC283003.1	[23]
VbA26	Human	UK	GU971638.1	[48]
	Kangaroo	Australia	KM366140.1	[31]
	Kangaroo	Australia	KU531700.1	[24]
	Rabbit	Australia	KU531701.1	[24]
	Rabbit	Australia	KU531705.1	[24]
	Rabbit	Australia	MG516797.1	[26]
	Human	New Zealand	MT265707.1	[55]
	River water and Riverbed Sediment	South Africa	-	[42]
VbA26R3	Rabbit	Spain	Acc Number	Present study

VbA26R4	Rabbit	Australia	HM852433.1	[22]
	Rabbit	Australia	KC283004.1	[23]
VbA27	Human	New Zealand	KY123920.1	[54]
	Human	New Zealand	MT265696.1	[55]
	Human	New Zealand	MT265697.1	[55]
VbA28	Human	UK	GU971631.1	[48]
	Rabbit	China	100% homology with GU971631.1	[16]
	Rabbit	Australia	MG516795.1	[26]
	Human	New Zealand	MT265708.1	[55]
	Riverbed Sediment	South Africa	-	[42]
VbA29	Rabbit	China	FJ262734.1	[12,21]
	Rabbit	China	GU097639.1	[13]
	Rabbit	China	GU097640.1	[13]
	Rabbit	China	GU097642.1	[13]
	Human	UK	GU971633.1	[48]
	Human	UK	GU971643.1	[48]
	Rabbit	China	100% homology with GU971633.1	[16]
	Rabbit	Australia	MG516793.1	[26]
	Riverbed Sediment	South Africa	-	[42]
	Rabbit	China	MZ460993.1	[]
	Rabbit	China	MZ460995.1	[]
VbA29R4	Human	Sweden	KU852734.1	[58]
VbA30	River water and Riverbed Sediment	South Africa	-	[42]
VbA31	Rabbit	China	MZ460998.1	[18]

	Rabbit	China	MZ460996.1	[18]
VbA31R4	Human	Greece	KU852732.1	[58]
VbA32	Human	UK	GU971646.1	[48]
	Rabbit	China	HQ397718.1	[14]
	Rabbit	China	100% homology with GU971646.1	[16]
	Riverbed Sediment	South Africa	-	[42]
VbA33	Human	UK	GU971635.1	[48]
	Rabbit	Egypt	MT328207.1	[29]
	River water and Riverbed Sediment	South Africa	-	[42]
	Rabbit	China	MZ460997.1	[18]
VbA34	Human	UK	GU971645.1	[48]
	Human	Spain	KU129016.1	[59]
	River water and Riverbed Sediment	South Africa	-	[42]
VbA35	Rabbit	China	GU097647.1	[13]
VbA36	Human	UK	GU971648.1	[48]
	Rabbit	China	GU097641.1	[13]
VbA37	Human	UK	GU971639.1	[48]
	River water and Riverbed Sediment	South Africa	-	[42]
VbA38	Human	Canada	-	[57]
